# Inter-Fraction Motion and Dosimetric Analysis of Volumetric Modulated Arc Therapy for Craniospinal Irradiation in Adult Medulloblastoma Patients

**DOI:** 10.3390/jpm14121134

**Published:** 2024-11-30

**Authors:** Ilaria Bonaparte, Fiorella Cristina Di Guglielmo, Federica Fragnoli, Rosilda Cuscito, Chiara Indellicati, Christian De Pascali, Alessia Surgo, Roberta Carbonara, Valerio Davì, Maria Annunziata Gentile, Roberto Calbi, Morena Caliandro, Giuseppe Sanfrancesco, Alberto Aga, Pietro Cardetta, Michele Antonicelli, Annarita Ciocia, Domenico Curci, Maria Paola Ciliberti, Alba Fiorentino

**Affiliations:** 1 Department of Radiation Oncology, Miulli General Regional Hospital, Acquaviva delle Fonti, 70021 Bari, Italy; ilaria.bonaparte@medipass.it (I.B.); f.diguglielmo@miulli.it (F.C.D.G.); a.surgo@miulli.it (A.S.); v.davi@miulli.it (V.D.); m.ciliberti@miulli.it (M.P.C.); a.fiorentino@miulli.it (A.F.); 2 Department of Medicine and Surgery, LUM University, 70010 Casamassima, Italy; 3 Department of Radiology, Miulli General Regional Hospital, Acquaviva delle Fonti, 70021 Bari, Italy

**Keywords:** radiotherapy, craniospinal irradiation (CSI), medulloblastoma, adults, brain tumors

## Abstract

**Background/Objectives**. Adult medulloblastoma (AMB) patients should receive postoperative craniospinal irradiation (CSI) as a standard treatment. Volumetric intensity-modulated arc therapy (VMAT) is a promising method for CSI. This report summarizes the repositioning and dosimetric data outcomes for six AMB patients. **Methods.** Complete CSI and posterior cranial fossa irradiation, or tumor bed boost irradiation with Linac-based VMAT, was performed and evaluated. Patients were immobilized in the supine position with two thermoplastic masks (head-neck and abdomen). To ensure inter-fraction reproducibility during radiotherapy (RT), a single cone-beam CT (CBCT) scan for each isocenter and real-time surface-guided RT using AlignRT^®^ were performed daily before and during the RT session. Match values of all three translational axes (x = lateral, y = longitudinal, z = vertical) were recorded. **Results**. From August 2022 to September 2023, six AMB patients were treated with CSI: three women and three men with a median age of 32 (22–42). All cases were classical MB, four were low risk, and two were defined as high risk due to the metastatic disease. All patients underwent surgery; two received a gross total resection. Low-risk patients received 36 Gy for CSI and a 54 Gy boost, while high-risk patients received 39 Gy for CSI. No significant toxicities greater than G2 were observed during RT, and only two cases reported decreased platelet counts. The dose to the organs at risk was low and acceptable. The mean dose to the heart, lungs, eyes, stomach, and thyroid were 4.4 Gy, 8.5 Gy, 12 Gy, 8.7 Gy, and 11 Gy, respectively. In terms of repositioning data, 124 CBCT scans were analyzed. Inter-fraction CBCT mean values for the study population in all translational directions were inferior to 2 mm in more than 90% of cases. **Conclusions**. VMAT is a convenient and effective treatment for AMB. Positioning and immobilization with masks (head and neck plus abdomen) reduce inter-fraction motion.

## 1. Introduction

Medulloblastoma is a cancerous tumor that develops from primitive neuroectodermal cells. It typically spreads through the cerebrospinal fluid and is primarily located in the posterior cranial fossa (PCF). This type of tumor accounts for about 20–30% of all intracranial neoplasms in children, with a median age of 5 years [1,2]. It is a rare tumor in adults, with an incidence of approximately 0.5 per 100,000 population per year [2,3,4,5].

If feasible, the recent ESMO guidelines for adult medulloblastoma (AMB) treatment recommend a gross total resection (GTR) in all patients. Otherwise, a maximal safe resection should be performed for children and adults [6].

According to the latest guidelines, undergoing post-operative cranio-spinal irradiation (CSI) within six weeks after surgery is essential. The guidelines suggest using advanced techniques such as helical tomotherapy (HT) or volumetric intensity-modulated arc therapy (VMAT) to perform CSI [7]. These modern techniques ensure adequate coverage of the target volume, minimizing the risk of future comorbidities associated with radiation exposure and allowing for the sparing of the organs at risk (OARs) to reduce the risk of further complications.

A recommended total dose of 36–39 Gy should be delivered in daily fractions of 1.6–1.8 Gy, 5 times per week for CSI. It is also necessary to escalate the dose locally to the primary cancer focus (PCF) and the tumor bed, with a total dose of up to 54–55.8 Gy, as indicated by reliable sources [2,6,7].

With conventional three-dimensional conformal radiotherapy (3D-CRT), CSI uses two opposing lateral craniocervical fields combined with a posterior spinal field. In taller individuals, two to three adjacent radiation fields may be used [7]. Ensuring a proper alignment between the lateral and posterior fields is essential to prevent the risks of over- or under-radiation therapy, which may lead to myelitis or local tumor failure. This match is typically achieved by adjusting for both the superior divergence of the posterior spinal field and the caudal divergence of the lateral fields.

Implementing a shifting junction strategy is a common practice to achieve a risk distribution of over- or under-dosage across the field matching. This technique aims to mitigate the potential negative impact of either scenario, which could lead to undesirable outcomes. By strategically shifting the junction points, the probability of such adverse effects can be minimized, resulting in a more optimal treatment plan and delivery [7]. CSI is usually performed with the patient in the prone position to visualize the field junction best, especially for children.

Technically, adult CSI cases are different and more complex than pediatric cases due to the length of the craniospinal axis (40–50 cm for children versus 65–75 cm for adults) and the greater body mass index of adults [7]. For these reasons, adult patients are usually in the supine position. 

New techniques and technologies, including volumetric modulated arc therapy (VMAT) and helical tomotherapy (HT), have been developed to overcome issues with junctions of several RT fields or non-target radiation doses delivered to the patient. 

VMAT has recently been introduced in clinical practice for several types of tumors, including brain tumors, prostate cancer, head and neck cancers, anal canal cancers, and cervical cancers, following intensive validation [7,8,9]. It allows for the delivery of the dose to the planning target volume (PTV) in a single gantry rotation using a volumetric approach. This RT technique aims to optimize the dose delivery and the gantry’s rotational speed. The goal is to achieve the desired dose distribution that improves conformity to the target while minimizing exposure to the healthy tissue surrounding the tumor [7]. For CSI, VMAT has made it possible to tailor the dose for better uniformity over the craniospinal axis, reducing the dose to organs at risk (OARs) and, consequently, the possibility of radiation comorbidities in the future [10,11]

Due to the rarity of the disease in adult patients, there needs to be more research on dosimetric data and inter-fraction reproducibility for CSI in AMB patients. 

This retrospective analysis addresses this gap by examining the treatment outcomes related to dosimetric data, inter-fraction reproducibility, and clinical outcomes in six AMB patients treated with VMAT-CSI and surface-guided radiotherapy (SGRT) [12].

## 2. Materials and Methods

Our Radiation Department started clinical activity in June 2019. It is equipped with two Linac (TrueBeam^TM^, Varian Medical Systems—Siemens) and two AlignRT^®^ as SGRT. Around 1300 cancer patients are treated annually and we started treating adults for CSI in August 2022. 

For the current analysis, patients over 18 years old, affected by histologically confirmed MB, and treated with linac-based CSI-VMAT were selected retrospectively. During RT, acute side effects were evaluated using the Common Toxicity Criteria (CTC) V5.0. Informed consent was obtained from all patients included in the study.

**Figure 1:**
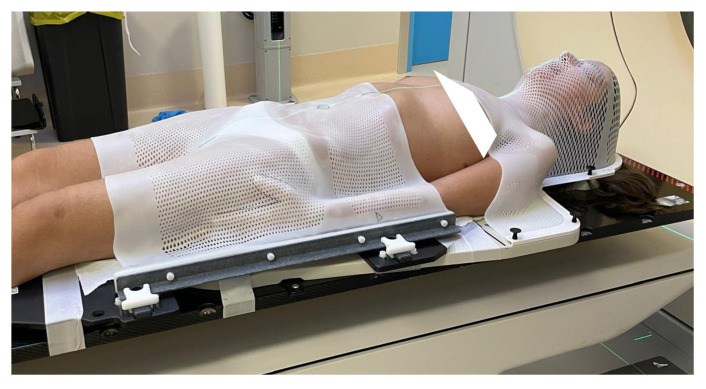
Immobilization system.

Patients were immobilized in the supine position using a head–shoulder (open-face) reinforced thermoplastic mask and a single sheet of thermoplastic that forms over the breast, thorax, abdomen, hip, or pelvis to aid patient positioning and reproducibility (Civco), as shown in Figure 1. A computed tomography (CT) simulation was performed without contrast, including the craniospinal axis, acquiring slices of 2 mm thickness.

The clinical target volume (CTV) included the entire brain (CTV brain) and the subarachnoid space of the neuraxis (CTV spinal). The planning target volume (PTV) was obtained from the CTV plus an isotropic margin of 5 mm in all directions for the CTV brain and a 5–10 mm margin for the CTV spine, as reported by other authors [7,10]. 

The heart, brain stem, chiasm, cochlea (bilateral), lacrimal gland (bilateral), eyes (bilateral), lens (bilateral), thyroid gland, humerus head (bilateral), duodenum, esophagus, bladder, bowel, kidneys (bilateral), larynx, lungs (bilateral), mandible, optic nerve (bilateral), oral cavity, ovaries (bilateral for female patients), parotid (bilateral), pituitary gland, rectum, stomach and submandibular glands (bilateral) were contoured as OARs. The prescription dose for CSI ranged from 36 Gy to 39 Gy in 1.6–1.8 Gy/fraction. A dose boost to the PCF or tumor bed was administered until a total dose of 50.4–54 Gy/28–30 fractions was reached. 

Target coverage was expressed as the PTV volume receiving at least 95% of the prescribed dose (V95%) and the volume receiving more than 107% of the prescribed dose (V107%). Some generally acceptable criteria were drawn here: maximal dose to lenses < 5–10 Gy; mean heart dose < 10 Gy; median dose to kidney lower than 20 Gy; mean lung dose should not be more than 15 Gy.

VMAT plans were generated with Eclipse (Varian Medical Systems—Siemens) for all six patients and delivered on a TrueBeam^TM^ linac (Varian Medical Systems—Siemens). 

All cases were treated with multiple isocenters due to the target length ranging from 51.6 cm to 74.5 cm. Fogliata et al. [7] reported that the first isocenter was localized in the brain; the second was located in the upper thoracic column (T3–T5), and the third was in the lumbar region. The position of the isocenters differs only in the craniocaudal direction. We ensured that the lateral and anterior-posterior coordinates were identical to minimize errors during manual shifts between the isocenter. Two arcs were used for each isocenter. Plans were optimized, defining at least a 5 cm region overlapping between arcs of different isocenters to guarantee a smooth dose transition and prevent a field match and hot dose.

The arcs in the brain region presented an avoidance sector of 120° (range 300°; −60°) to limit the irradiation of the optical structures (lens, eye, optical nerve). The arcs in the thoracic and lumbar region also included an avoidance area based on the contouring of the arms with an additional 2 cm margins. The area has two benefits: it avoids arm positioning issues and limits lung exposure.

Dose uniformity was evaluated using the dose homogeneity index (DHI). DHI = D5%/D95%; D5% was the irradiation dose that 5% of the PTV received, and D95% was the irradiation dose that 95% of the PTV received [13]. A DHI value of 1 suggested better dose uniformity, and dose conformity was evaluated with the conformity index (CI). A CI close to 1 indicated better conformity [13]. 

During RT, to evaluate inter-fraction reproducibility, IGRT with a single cone-beam CT (CBCT) scan for each isocenter and real-time SGRT were performed daily before and during the RT session [14,15]. 

Before treatment, the Radiation Therapists (RTTs) adjusted patient positioning using SGRT. After this procedure, the first CBCT scan verified that the patient’s position was correct, accepting a threshold for acceptable motion inferior to 5 mm. No rotational corrections were allowed. CBCT match values of all three translational axes (x = lateral, y = longitudinal, z = vertical) were recorded.

Statistical analysis was performed with the Statistical Package for Social Sciences (SPSS, Chicago, IL, USA) software package, version 18.0, for Windows. A single-factor analysis of variance was performed. This study considered a two-tailed *p* value < 0.05 statistically significant. The interclass correlation coefficient (ICC) was used to describe how strongly units in the same group resemble each other.

## 3. Results

From August 2022 to September 2023, six AMB patients were treated with CSI: three women and three men with a median age of 32 years (22–42). All cases were classical MB, four were low risk, and two were defined as high risk due to the metastatic disease. All patients received surgery; in two cases, a GTR was performed. 

After 30 days from surgery, all patients underwent craniospinal MRI to evaluate the presence of residual disease or metastases. RT started six weeks after surgery (range 6–9 weeks) based on the clinical condition of the patients. A total of six patients underwent CSI treatment. Among them, two patients received 39 Gy and four received 36 Gy. During RT, no acute toxicities more than G2 (mild toxicities) were documented; only two cases reported a reduction in platelet counts without interrupting the treatment, and two patients suffered from grade 2 nausea and vomiting the first three days of RT, solved with metoclopramide. At the end of RT, mild alopecia was reported in all patients. No patients received chemotherapy before or concomitantly to RT. All patients received chemotherapy at the end of RT.

All patients are alive and were without disease at a median follow-up of 10 months (range 6–18 months). The patients with residual disease or metastases had negative MRI scans 8–10 months after the end of RT. No late side effects related to RT were documented.

**Figure 2:**
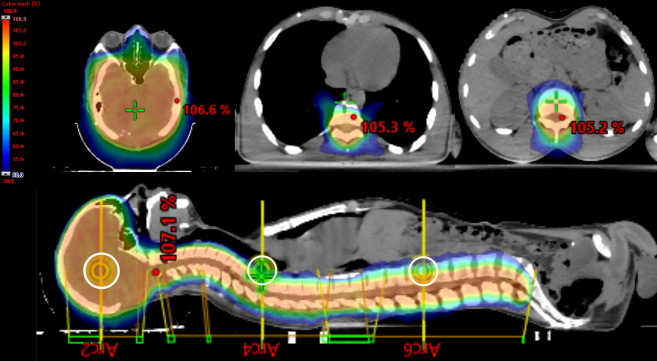
Sagittal views and transversal views. Color wash range: 50% to 107%. The white circle represents the three isocenters of the CSI plan.

Regarding the RT plan, Table 1 and Table 2 report all dosimetric data, while Figure 2 presents the dose distributions in the sagittal and transversal views. 

Regarding the geometry of the RT plans, for CSI, five patients out of six received an RT plan with three isocenters, with two arcs for each isocenter and a mean distance between isocenters of 25 cm (range 20–27 cm).

The mean PTV length and PTV volume were 69 cm (range 51.5–75.3 cm) and 2324.1 cc (range 803–3451 cc), respectively.

The mean V95% for PTV (% prescription dose delivered to 95% percent of volume) was 98.4% (range 97.4–99.0%), and the mean D1% (irradiation dose delivered to 1% of volume) was 38.3 Gy (range 37.0–41.7 Gy). 

In terms of indices, the mean CI and DHI were 0.90 and 1.06, respectively (Table 1). The target volume exposed to a high dose (V107%) in VMAT was inferior to 0.05% (range 0–0.04%), and the mean monitor units were 1167 (range 757–1415). The mean treatment time was 30 min (range 20–50).

The Dmean of OARs was reported as 4.4 Gy for the heart, 8.6 Gy for the lungs, and 6.8 for the kidneys (Table 2).

The study analyzed 124 CBCT scans to determine the accuracy of repositioning data. Table 3 reports the mean values for inter-fraction CBCT in all translational directions. As shown in Table 4, the inter-fraction motions for all patients were less than 2 mm for over 90% of the fractions, and 99% were less than 5 mm for 99% of the fractions. Based on the present results, the margin from the CTV to the PTV could be evaluated to be reduced for spinal tumors from 10 mm to 5–6 mm to avoid doses to OARs.

A review of all dosimetric data on CSI in adults was conducted and is reported in Table 5.

## 4. Discussion

MB is a rare disease that shares similarities and differences with its childhood counterpart. In the pediatric population, the treatment of medulloblastoma is well defined and consists of maximal surgical resection, CSI, a boost to the posterior fossa, and chemotherapy. The 5-year progression-free survival (PFS) rate for many MB cancer patients is 75–85% [2]. 

Due to the low incidence of the disease in adults, limited data on treatment and prognosis are available. The available data mainly consist of retrospective studies describing a highly diverse array of treatment strategies. However, CSI is necessary at all stages of MB, including in adults, for both curative and palliative treatments [1,2,3,4]. 

Until several years ago, the standard approach was 3D-CRT, which required a matched junction between the cranial and spinal fields. However, this approach can lead to inadequate treatment outcomes due to the likelihood of over- or under-dosing irradiation to the central nervous system [18,19]. Emerging technologies and techniques in radiation therapy, such as VMAT or HT, have proven to be more effective solutions to overcome this issue. A recent retrospective analysis published by Öztunali et al. [20] assesses the toxicity profile of different radiation techniques and estimates survival rates in medulloblastoma patients. In total, 43 courses of CSI and three local RT course were administered to the 46 patients; 30 were male, and the median age was 7 years (range 1–56). Only five patients were aged more than 18 years old. A median total RT dose of 55 Gy (range 44–68) and a median CSI dose of 35 Gy (range 23.4–40) were delivered. Patients were immobilized in the supine position with thermoplastic masks for head support and vacuum cradles for body support and anesthetized if needed. Regarding the radiation technique, the 5-year EFS rate in the 3D-CRT group was higher compared to the HT group (93% vs. 67%).

Other analyses confirmed the latter data, reporting that modulated irradiation techniques should be preferred to 3D-CRT because they are associated with a lower risk of side effects [7,21]. Our analysis reported similar results. All patients completed CSI without interruptions, and no patients reported high-grade acute toxicities. Only mild nausea and vomiting were documented, which were resolved with antiemetic drugs. Indeed, in terms of late toxicity, evaluating our patients is impossible due to the shorter follow-up (median 10 months). Still, to date, no late toxicity related to RT has been documented.

Regarding technical issues, CSI is a complex technique that significantly impacts long-term patients’ quality of life and potential side effects. 

Advanced techniques, such as VMAT or HT, reduce uncertainties related to tumor shape, delivering optimal conformal RT while minimizing integral dose. In adult patients, new techniques offer the advantage of replacing junctions and matching lines with overlapping arcs. The optimization system can automatically account for the dose contribution. Overlapping arcs can reduce residual errors due to couch shift movements between isocenters. As a result, VMAT or HT is preferred for CSI.

Patient position verification varied substantially among published studies. No data on inter-fraction motion were reported, and different immobilization systems were used in adults. In their analysis, Fogliata et al. positioned two patients in the prone and two in supine positions. Only a head-neck mask was utilized in the supine position [7]. Balducci et al. used the prone position with a thermoplastic mask for the head, while other authors used a head mask and a body VAC-Lok Cushion system [10,20,21].

To our knowledge, data about inter-fraction motion for adult CSI have yet to be reported in the literature. In the present analysis, the study of 124 CBCT scans found that this immobilization (head, neck, and abdomen masks) system produced acceptable intra-fraction motion. The motion was less than 5 mm for over 99% of fractions. Nevertheless, the ICC is under 0.4, reflecting that the reliability is poor and the magnitude of movement is shallow. Inter-fraction motions were less than 2 mm for about 93% of the fractions and less than 1 mm for over 80% of cases. 

The latter results show that a 6–7 mm margin from the CTV to the PTV is sufficient for CSI treatment using head-neck and abdomen thermoplastic masks. These data can help develop an alternative AMB immobilization system involving head-and-neck masks or vacuum locks.

In theory, with multiple CBCTs, the entire patient length can be scanned for proper positioning before treatment; the adopted imaging modality, verifying all isocenters before starting therapy, is an easily implementable code of practice. Beam delivery should be enabled only if the image-based verification results are acceptable for all isocenters. The initial patient setup accuracy typically ranged between 5 and 10 min for the present patients, including the total time needed to perform the IGRT procedure with CBCT and optical surface monitoring, couch displacement foreseen in the treatment plan, and the evaluation and decision processes.

Beam-on time ranged from 3 min for the two whole arcs arrangement to 10 min for the six arcs for CSI. The time patients spent in treatment varied depending on the patient and the treatment session. During the first week of RT, the process was more complex for the Radiation Therapist Team, and the treatment lasted about 40–45 min. However, in the following weeks, the time required for treatment was reduced to 20–25 min. These values could be compared with the pure beam-on time for HT CSI treatments, which ranges from 10 to 16 min [7,17,22].

In terms of VMAT geometry, the number of isocenters was a function of three factors: (1) cranio-caudal PTV length, (2) field size in the y direction, and (3) adjacent field overlap of 5–10 cm. All patients in this report received multiple isocenter VMAT plans (median three isocenters) with a median distance of 25 cm and two arcs for each isocenter. The same data are reported by other authors [7,10,16].

Despite the sample size limitation (presented in all adult studies due to the rarity of the disease), our dosimetric findings align with those published by other authors [7,13,14,15]. These authors showed good target coverage and substantial sparing of OARs in the direct vicinity of the PTV. Table 5 compares VMAT, 3D-CRT, and HT dosimetric data published data on adult MB cases, showing similar results [7,10,22]. 

Fogliata et al. reported the dosimetric results of five studied cases (four were adults) treated with VMAT-CSI with 36 Gy in different European institutions using various planning approaches, showing that VMAT techniques achieved highly conformal treatment plans [7]. The mean doses to the lenses, lungs, heart, and kidneys were, respectively, 7.6 ± 1.6, 6.6 ± 3.0, 5.7 ± 2.3, and 6.1 ± 2.1 Gy, keeping those values acceptably low. 

Zong-wen evaluated the HT, VMAT, and 3D-CRT dose for five cases of AMB patients treated with 30–36 Gy via HT. The dose uniformity and conformity of HT and VMAT were good. The target volume exposed to a high dose (V107%) in VMAT was more significant than that of HT, and 3DCRT took the highest V107%. 

Our data showed different results in terms of V107%. Our mean value was 0.035%, compared to the values reported by Zong-wen et al., which fluctuated between 2.28% and 20.62%.

Regarding dose uniformity and conformity, the data are suitable for VMAT; DHI values were close to 1, and the CI values fluctuated between 0.9 and 1.02 with a mean value of 0.94, similar to those reported in the literature also with HT [16].

## 5. Conclusions

“CSI” refers to radiation planning and delivery, which is considered one of the most challenging processes in RT. The small sample size in all published studies on AMB patients limits the ability to determine which technique is superior. The six cases analyzed and treated with VMAT showed that this technique is effective and advantageous in treating adult patients with MB. However, the data from 124 cone beam CT (CBCT) scans demonstrate that the immobilization system is essential for reducing inter-fraction motion. 

In conclusion, new technology or techniques for CSI in AMB patients are statistically superior to 3D-CRT for reducing doses to organs at risk and achieving low-dose spillage.

## Figures and Tables

**Table 1:** Dose parameters for analyzed patients.

Category	Patient 1	Patient 2	Patient 3	Patient 4	Patient 5	Patient 6
**Dose prescription**	39 Gy	36 Gy	39 Gy	36 Gy	36 Gy	36 Gy
**V95%**	98.2%	99.5%	98.5%	99.0%	98.0%	97.5%
**V107%**	<0.05%	<0.05%	<0.05%	<0.05%	<0.05%	<0.05%
**D1%**	38 Gy	37.904 Gy	41.72 Gy	37.91 Gy	37.30 Gy	37 Gy
**D99%**	32.96 Gy	34.948 Gy	37.42 Gy	34.82 Gy	32.57 Gy	33.60 Gy
**Dmean**	36.76 Gy	36.69 Gy	40.54 Gy	36.70 Gy	36.00 Gy	36.00 Gy
**DHI**	1.09	1.04	1.1	1.05	1.07	1.06
**CI**	0.94	1.02	0.93	0.93	0.90	0.92
**PTV length cm**	74.5 cm	71.9 cm	75.3 cm	69.6 cm	70.6 cm	51.6 cm
**PTV volume**	3451.28 cm^3^	2052.32 cm^3^	2678.1 cm^3^	2610.31 cm^3^	2349.42 cm^3^	803.2 cm^3^
**Arc arrangement**	180.1–179.9° 179.9–180.1°	180.1–179.9° 179.9–180.1°	180.1–179.9° 179.9–180.1°	180.1–179.9° 179.9–180.1°	180.1–179.9° 179.9–180.1°	180.1–179.9° 179.9–180.1°
**Collimator rotation**	10–90° 5–355° 5–355°	10–90° 5–355° 5–355°	10–90° 5–355° 5–355°	10–90° 5–355° 5–355°	10–90° 5–355° 5–355°	5–355° 5–355°
**Number of isocenters**	3	3	3	3	3	2
**Arcs per isocenter**	2	2	2	2	2	2
**Distance between isocenter**	27 cm	27 cm	26 cm	25 cm	25 cm	20 cm
**Monitor units**	1175.5	1254.3	1036.5	1415.1	1364.9	757

Vn%: n% prescription dose delivered to percent volume; D1%: irradiation dose delivered to 1% volume, representing maximum dose; D99%: irradiation dose delivered to 99% volume, representing minimum dose; Dmean: average irradiation dose; CI: conformity index; dose homogeneity index (DHI).

**Table 2:** Irradiation dose of OARs: mean (SD).

	Category							
OAR	D1%	Dmean	V5%	V10%	V20%	V30%	V40%	V50%
Left lens	12.0 (5)	9.2 (5.4)	11.8 (5)	11.6 (5)	11.5 (5)	11.3 (4.9)	11.2 (4.8)	11.0 (4.8)
Right lens	12.0 (5)	9.3 (4.6)	11.9 (5)	11.7 (4.9)	11.5 (4.8)	11.3 (4.9)	11.3 (4.7)	11.2 (4.7)
Left eye ball	21.4 (8.4)	12.8 (6.2)	19.0 (8)	17.5 (8)	15.6 (7.5)	14.2 (6.9)	11.0 (6.7)	10.3 (7.8)
Right eye ball	20.7 (9.1)	11.8 (5.8)	17.8 (8.5)	15.9 (7.5)	13.7 (6.7)	12.4 (6.1)	11.6 (5.8)	11.0 (5.5)
Left lung	24.8 (4.2)	8.3 (1.2)	18.0 (2.7)	14.5 (2)	11.5 (1.4)	9.8 (1.2)	8.7 (1.2)	7.5 (1.3)
Right lung	28.0 (3.2)	8.9 (1)	22.4 (2.2)	18.2 (1.5)	13.5 (0.9)	11.2 (0.8)	9.8 (0.9)	8.9 (0.9)
Heart	11.7 (3.6)	4.4 (1.2)	9.1 (3.1)	7.5 (2.6)	5.7 (1.8)	4.6 (1.3)	4.0 (1.1)	3.4 (0.9)
Liver	19.4 (2.8)	8.8 (1.6)	15.9 (2)	14.0 (1.6)	11.8 (1.4)	10.5 (1.5)	9.4 (1.6)	8.5 (1.7)
Stomach	14.9 (1.6)	8.7 (2)	13.1 (1.6)	12.0 (1.8)	10.8 (2)	9.8 (2.1)	9.0 (2.3)	8.3 (2.3)
Left kidney	18.1 (1.8)	6.5 (1.6)	14.7 (2.6)	12.2 (2.8)	9.4 (2.9)	7.7 (2.7)	6.4 (2.2)	5.3 (1.6)
Right kidney	17.2 (3.1)	7.1 (2.5)	14.4 (3.7)	12.7 (3.7)	10.6 (4)	9.0 (3.9)	7.7 (3.5)	6.5 (2.3)
Thyroid	18.7 (9)	11.0 (6)	16.7 (8.5)	15.2 (8)	13.4 (7.3)	12.0 (6.9)	11.2 (6.4)	10.3 (6)

Vn%: n% prescription dose delivered to percent volume; D1%: irradiation dose delivered to 1% volume, representing maximum dose; Dmean: average irradiation dose; SD (standard deviation); OARs: organs at risk.

**Table 3:** Inter-fraction motions (cm).

Patient	x (cm)	y (cm)	z (cm)	xyz (cm)
1	0.03 ± 0.31	0.01 ± 0.19	0.01 ± 0.08	0.29 ± 0.23
2	−0.08 ± 0.16	0.00 ± 0.14	−0.37 ± 0.17	0.44 ± 0.15
3	−0.02 ± 0.10	−0.25 ± 0.24	−0.04 ± 0.16	0.34 ± 0.20
4	−0.01 ± 0.09	−0.21 ± 0.09	−0.03 ± 0.12	0.25 ± 0.09
5 *	−0.18 ± 0.19	−0.19 ± 0.26	−0.07 ± 0.33	0.49 ± 0.19
6 *	0.07 ± 0.11	−0.17 ± 0.16	−0.07 ± 0.19	0.31 ± 0.11

Mean ± standard deviation. * Obese patient.

**Table 4:** Analysis of inter-fraction motions.

	Mean ± SE	ICC	<0.10 cm (%)	<0.20 cm (%)	<0.50 cm (%)
x (cm)	0.034 ± 0.033	0.142	80.7 (%)	91.1 (%)	99.2 (%)
y (cm)	−0.135 ± 0.042	0.195	87.1 (%)	96.0 (%)	99.2 (%)
z (cm)	−0.096 ± 0.051	0.279	86.3 (%)	92.7 (%)	99.2 (%)

SE = stadard error ICC: interclass correlation coefficient.

**Table 5:** Dosimetric data for VMAT, 3D-CRT, and HT.

	This Study	Fogliata et al. [7].	Zong-wen et al. [16]	Zong-wen et al. [16]	Zong-wen et al. [16].	Parker et al. [17]
Technique	VMAT	VMAT	VMAT	3D-CRT	HT	HT
Dose prescription	36–39 Gy	36 Gy	30–36 Gy	30–36 Gy	30–36 Gy	36 Gy
Heart	4.4	6.7	6.85	5.4	3.9	11
Kidneys	7.0	7.2	3.55	3.8	6.15	6.5
Lungs	8.5	7.5	4	5	4.2	6
Liver	8.8	7.1	4.42	5.01	4.96	8
Thyroid	11.0	11.9	-	-	-	25

VMAT: volumetric modulated arc therapy; 3D-CRT: three dimensional conformal radiotherapy; HT: helical tomotherapy.

## Data Availability

The data are available upon request from the corresponding author.

## Abbreviations

Posterior cranial fossa (PCF)
Adult medulloblastoma (AMB)
Gross total resection (GTR)
Craniospinal irradiation (CSI)
Helical tomotherapy (HT)
Volumetric intensity-modulated arc therapy (VMAT)
Three-dimensional conformal radiotherapy (3D-CRT)
Surface-guided radiotherapy (SGRT)
Common Toxicity Criteria (CTC)
Organs at risk (OARs)
Clinical target volume (CTV)
Dose homogeneity index (DHI)
Conformity index (CI)
Progression-free survival (PFS)
Cone-beam computed tomography (CBCT)
Planning target volume (PTV)
Vn%: n% prescription dose delivered to percent volume
D1%: irradiation dose delivered to 1% volume, representing maximum dose
Dmean: average irradiation dose
SE: standard error
ICC: interclass correlation coefficient
SD: standard deviation
RTT: Radiation Therapist
